# Linear Epitope-Mediated Immunological Cross-Reactivity in Livestock Product Allergens

**DOI:** 10.3390/ijms27041628

**Published:** 2026-02-07

**Authors:** Pengmei An, Yanjun Cong

**Affiliations:** College of Food and Health, Beijing Technology and Business University, Beijing 100048, China; an18701249218@163.com

**Keywords:** livestock products, allergens, linear epitopes, cross-reactivity

## Abstract

Linear epitopes, comprising continuous amino acid sequences, are critically implicated in the immunological cross-reactivity of livestock products owing to their stability during food processing. This paper aims to comprehensively review linear epitope-mediated cross-allergic reactions in major livestock products, including milk, eggs, and meat. Furthermore, this review systematically delineates the pathophysiological mechanisms underlying classical cross-allergic reactions, including α-Gal syndrome and avian egg syndrome, while addressing novel cross-reactive allergens, such as the thermostable poultry meat allergen Gal d 7 and the egg–milk cross-reactive protein α-vitellin, alongside their respective linear B-cell epitope profiles. Finally, this study summarizes the roles of these cross-reactive allergens in precise diagnosis and targeted therapy, providing a theoretical basis for future in-depth research on food cross-allergenic mechanisms.

## 1. Introduction

Food allergies have emerged as a global public health concern owing to their increasing incidence. Data from epidemiological studies on food allergies published in China between 2000 and 2021 indicate that food allergies affect about 5% to 12% of the country’s total population, showing an increasing trend over the years. The prevalence is highest among school-aged children (7–14 years), accounting for about 7–14% of this age group, followed by adults (2–11%) and infants (4–8%) [1]. In Western countries, adverse immune reactions to food impact about 5% of young children and 3–4% of adults, with prevalence rates continuously rising [2]. Among various food allergens, those from livestock products, such as meat, eggs, and milk, are particularly important considering their regular consumption by the general public worldwide. Reportedly, meats from various sources account for about 0.3–8% of all food allergies, depending on studies and regions [3], whereas milk and eggs are among the most common allergens in infancy and early childhood (global egg allergy prevalence in children: approximately 6–8%) [4]. Moreover, the estimated prevalence of cow’s milk allergy (CMA) has been reported to be 0.5–3% by the age of 1 year in developed nations [5].

Allergic reactions to livestock products are predominantly mediated by the immunoglobulin IgE. Upon entering the body, an allergen’s epitopes are recognized by the immune system, triggering a cascade of responses [6]. Antigenic epitopes can be categorized into B- and T-cell epitopes. Based on their spatial structures, B-cell epitopes, which are directly recognized and bound by IgE, are further classified as conformational or linear epitopes. Linear B-cell epitopes comprise continuous amino acid sequences in the primary structure, whereas conformational B-cell epitopes rely on the protein’s native three-dimensional (3D) structure. As a result, conformational and linear B-cell epitopes are also known as discontinuous and continuous B-cell epitopes [7]. Importantly, while the majority of B-cell epitopes are conformational, linear epitopes play a unique role in allergies to livestock products. This is because these products often undergo processing, such as heating, cooking, fermentation, or enzymatic hydrolysis, which disrupts higher-order protein structures and inactivates conformational epitopes [8]; in contrast, linear epitopes retain their immunoreactivity post-processing. Consequently, identifying heat-stable, processing-resistant epitopes is a key focus in livestock allergen research [9,10].

**Figure 1:**
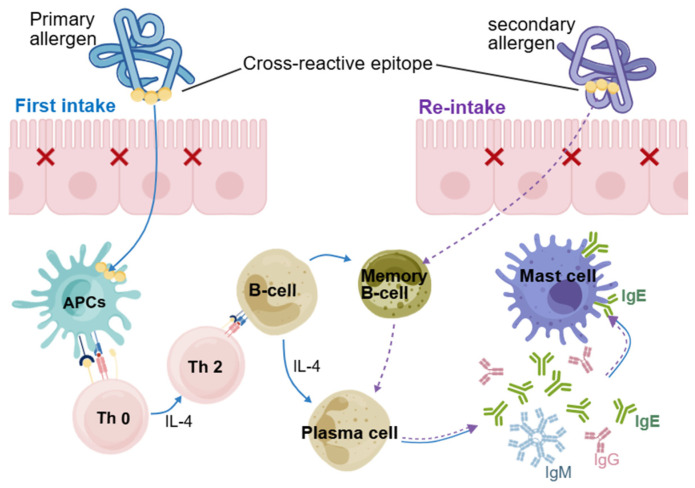
Simplified schematic diagram of cross-reactive linear epitopes involved in cross-allergic reactions. APCs, antigen-presenting cells; Th0, naive T helper cells; Th2, type 2 T helper cells.

Notably, linear epitopes form the molecular basis for mediating allergy-associated cross-reactivity, which occurs when sensitization to a primary allergen leads to the elevation of specific IgE antibodies, the activation of immune cells (such as T cells, mast cells, or basophils), and subsequent allergic reactions in a patient. Shared regions between two different allergens, known as cross-reactive epitopes, enable this process (Figure 1); multiple similar epitopes may exist in both primary and secondary allergens [11].

This review focuses on three major livestock products, namely, meat, eggs, and milk, to systematically summarize research progress on linear epitope-mediated immunological cross-reactivity of corresponding key allergens. Furthermore, advances in understanding linear epitope-mediated intra- and inter-species cross-reactions have been comprehensively discussed. Additionally, this study evaluated the current applications and prospects of diagnostic techniques and therapeutic strategies for livestock product allergies. The findings of this review may offer valuable insights for further research and clinical practice related to allergies.

## 2. Advances in the Cross-Reactivity of Animal Products

### 2.1. Advances in Immunological Cross-Reactivity in Meat

#### 2.1.1. Cross-Reactivity Among Different Meat Types

Compared with other food allergens, cross-reactivity among different meats, particularly red meat, is less common. Although clinically relevant reactivity between meats often occurs, it rarely progresses to sensitization against meat-specific epitopes [12]. Serum albumin is the primary allergen driving cross-reactivity among mammalian meats and associated with primary beef allergy, bird-egg syndrome, and cat-pork syndrome [13]. This protein exhibits high amino acid sequence homology across species (sequence identity among mammals: >70%; with chickens: <50%), resulting in similar linear B-cell epitopes [14]. To date, serum albumins from various species have been well characterized [15]. Reportedly, serum albumin has been shown to mediate cross-reactivity between cow milk and beef, with multiple proteins being shared between both products; bovine serum albumin (BSA; Bos d 6) and immunoglobulins (Bos d 7) are the main contributors to allergic reactions [16].

Primary allergy to poultry meat is also uncommon and typically limited to avian species [17]; nevertheless, individuals sensitized to chicken often show mild reactions to other poultry meats, such as turkey, pigeon, and quail [18]. Recently, Klug et al. [18] identified a novel heat-stable chicken allergen, Gal d 7, which showed >95% identity for amino acid sequence among chicken, duck, goose, and turkey in the Myosin light chain sequence. Western blotting further confirmed the cross-reactivity of recombinant Gal d 7 among chicken, duck, goose, and turkey. Notably, poultry meat cross-allergy can also manifest as fish-chicken syndrome [19] or bird-egg syndrome. Chicken serum albumin (Gal d 5) is a well-established major allergen that exhibits substantial cross-reactivity in chicken meat [20].

**Figure 2:**
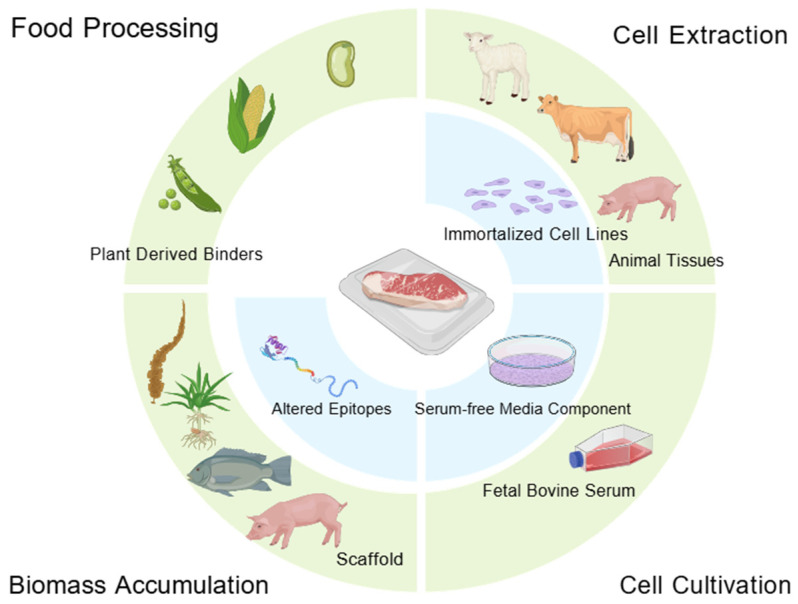
Cross-reactivity risks involved in the cultured meat production process; green, currently known allergens; blue, potential unknown allergens.

Cultured meat, an emerging technology that produces meat via cell culture, presents notable promise as a high-quality, safe, and stable animal protein source [21]. However, it still poses a significant risk of cross-reactivity, considering that the production process may involve biological materials from other species, including soy [22], fish [23], and gelatin [24] scaffolds (Figure 2). Furthermore, immortalized cell lines can harbor novel, uncharacterized allergens [25]. Consequently, even with comprehensive allergen labeling, consumers may not fully avoid sensitization risks, as these novel allergens remain uncontrollable. Ultimately, consuming cell-cultured meat could lead to the ingestion of unknown allergens, potentially affecting even those without a prior history of meat allergy. Therefore, the widespread adoption of cultured meat requires further technological validation.

#### 2.1.2. α-1,3-Galactose (α-Gal) Syndrome (AGS)

**Figure 3:**
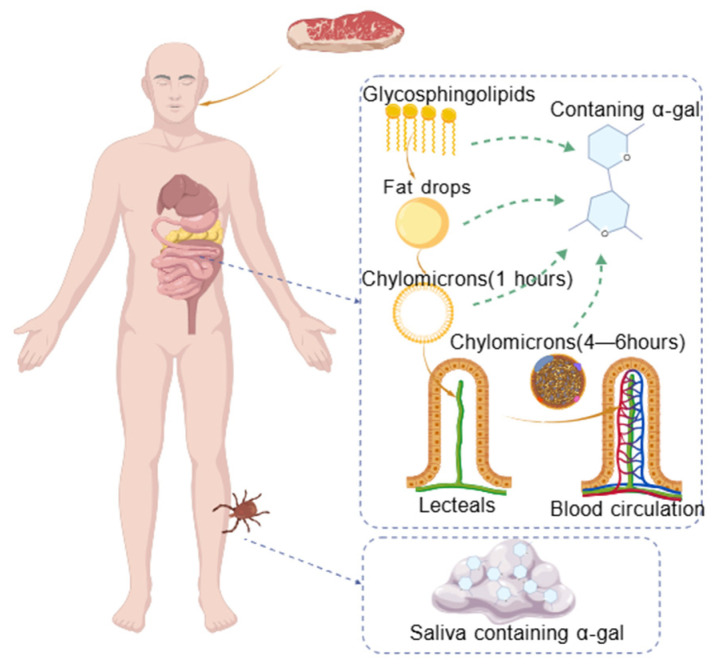
Schematic diagram of the hypothesized digestion process of glycosphingolipids containing the α-1,3-galactose epitope in vivo.

AGS is one of the classic examples of meat cross-reactivity [26]. Its molecular basis stems from the identical α-Gal epitope structure in red meats from different mammals, enabling specific IgE antibodies to recognize this epitope across species and triggering broad allergic reactions in mammalian red meat. The α-Gal epitope is a trisaccharide structure of Galα1-3Galβ1-4GlcNAc-R that is attached to proteins or lipids. This continuous glycosylation sequence is recognized by B-cells, conforming to the definition of a linear epitope. It does not rely on the three-dimensional folding structure of proteins and therefore does not belong to the conformational epitope. Reportedly, AGS onset strongly correlates with prior tick bites, which disrupt natural tolerance to red meat in humans [27]. Consequently, individuals without previous red meat allergy histories can develop severe allergic reactions post-consumption. After ingesting mammalian red meat, patients typically experience delayed allergic reactions by 3–6 h, ranging from urticaria and gastrointestinal distress to severe anaphylaxis [28]. A leading hypothesis attributes this delay to α-Gal epitopes ingested as glycosphingolipids. Because glycosphingolipids cannot directly bind to IgE antibodies, the reaction usually occurs after the breakdown of glycosphingolipids into chylomicrons carrying exposed α-Gal epitopes, which initiates the immune cascade [29,30]. Schematically, post-consumption, glycosphingolipids form chylomicrons within 1 h, which traverse the intestinal wall into lacteals, undergo size reduction over 4–6 h, and enter systemic circulation (Figure 3). The exposed epitopes then cross-link with specific IgE antibodies, thereby triggering allergic reactions.

Based on this hypothesis, most studies on AGS have focused on lipids. Although previous studies have suggested that only the lipid-bound α-Gal epitope induces effector cell activation [31], recent research [32] has shown that protein-bound α-Gal can activate effector cells. Reportedly, α-Gal epitopes on BSA impede its transport across intestinal Caco-2 monolayers, causing epitope-containing peptides to accumulate within intestinal cells. This represents a promising new avenue for exploring the sensitization mechanisms of the α-Gal epitope. Sharma et al. [33] used nanospray ionization mass spectrometry to investigate α-Gal-bound lipids in Amblyomma ticks by conjugating α-Gal to lipids and proteins for basophil activation tests. They reported that both forms activated granulocytes, confirming their sensitizing potential. Furthermore, Hils et al. [34] demonstrated that α-Gal-carrying glycosphingolipids alone could trigger allergic reactions in an AGS mouse model, with interleukin IL-4 playing a crucial role in α-Gal-specific IgE antibody production following tick bites. Overall, these results indicate that the glycosphingolipid digestion hypothesis does not fully explain α-Gal sensitization, warranting further investigation.

Recent efforts have focused on identifying potential α-Gal lipid and protein epitopes in the salivary glands and digestive tracts of ticks [35,36,37]. For example, Valcárcel et al. [38] reported a >250 kDa protein that contains the α-Gal epitope in tick salivary glands and digestive tracts. Similarly, Ristivojevic et al. associated the sensitizing potential of digesta with larger peptide fractions [32]. Collectively, these findings highlight the correlation between α-Gal epitope sensitization and larger peptide structures.

### 2.2. Advances in Immunological Cross-Reactivity in Egg

Among individuals with egg allergies, about 10% are sensitized to five of the six major egg allergens (Gal d 1–6), with cross-reactivity between yolk allergens (Gal d 5 and Gal d 6) and the egg white allergen Gal d 1 occurring in about 30% of diagnosed cases [39]. Clinical cross-reactivity is common among avian egg proteins (e.g., from hen, turkey, duck, gull, and quail), as reported in multiple studies [40,41,42]. Ovomucin (OVM), a highly heat-stable allergen, has been shown to drive reactions in cooked eggs [43]. For example, Lee et al. [44] reported that OVM remained stable from room temperature to 120 °C, maintaining cross-reactivity between cooked hen eggs and quail eggs.

**Figure 4:**
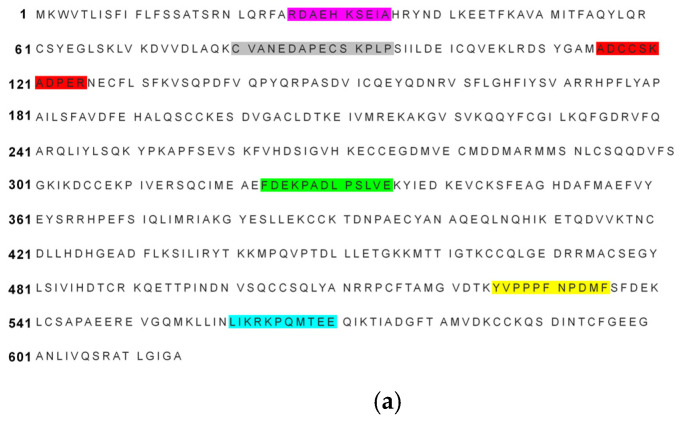

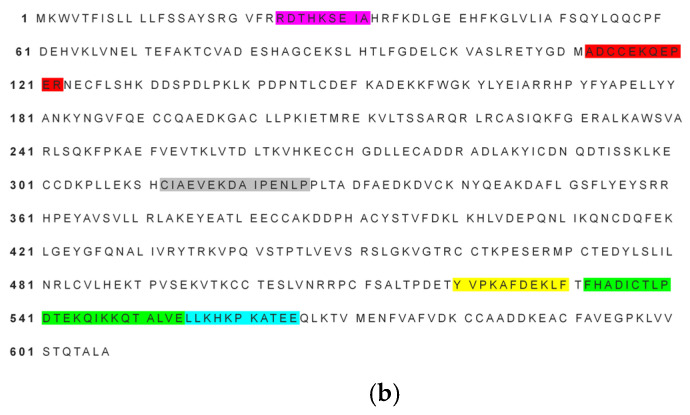
Egg–milk cross-reactive epitopes. The identical color markings in figures (**a**,**b**) represent a pair of cross-reactive epitopes. (**a**) Cross-reactive epitopes on BLAST2.13.0-predicted α-vitellin. UniProtKB/Swiss-Prot: P19121.2. (**b**) Cross-reactive epitopes on BLAST-predicted bovine serum albumin. GenBank: CAA76847.1.

Research on yolk allergens remains limited, likely due to challenges in the purification of allergens. Cross-reactivity involving the yolk allergen Gal d 5 has been reported to cause bird-egg syndrome through reactions to airborne avian allergens, such as feather dust [20]. Zhang et al. [45] reported a 40.9% co-positivity rate for egg sIgE among milk sIgE-positive children and 44.3% for milk sIgE among egg sIgE-positive children from Southern China, suggesting an association between egg and milk allergies. In 2021, a bioinformatics study predicted cross-reactive epitopes between cow milk and eggs, revealing 44.065% sequence similarity between α-vitellin and BSA, including similar 6 sequences (Figure 4). In this study, relevant sequences were synthesized via the Fmoc solid-phase synthesis method, and their allergenicity was identified via ELISA and WB. Finally, the cross-allergic epitope sequences on α-vitellin are as follows: C_312_IAEVEKDAIPENLP_326_, F_525_DEKLFTFHADICTLPDTEKQIKKQTALVE_554_, Y_520_VPKAFDEKLF_530_, and L_555_ LKHKPKATEE_565_ [46]. However, no other cross-reactive allergens have been reported to date, and animal studies are lacking, underscoring the need for further research to elucidate egg-cow milk cross-reactivity.

### 2.3. Advances in Immunological Cross-Reactivity in Milk

Reportedly, a commercial detection kit for β-lactoglobulin (BLG) [47] has shown extensive cross-reactivity among mammalian milk. Phylogenetically related mammals, such as cows, sheep, and goats, express highly homologous milk proteins (>82% identity), leading to a high risk of cross-allergy and further emphasizing the role of protein homology. A study reported that only 8% and 4% of patients with CMA react to donkey and horse milk, respectively, owing to lower homology [48]. BLG is a key allergen responsible for cross-reactivity among dairy products. Its absence in camel milk has been shown to prevent cross-reactivity with cow milk [49]. Xie et al. [50] developed a novel detection kit targeting BLG cross-reactive epitopes BLG-1 (KPTPEGDLEILLQK), BLG-2 (LQKWENDECAQKKIIAEKTKIPAVFKIDA), and BLG-3 (KALKALPMHIRLSFN). Notably, this kit demonstrated high specificity, for example, when tested for goat milk against goat, horse, donkey, and camel milk, only goat milk was detected by the kit, indicating effective improvement in the detection threshold and precision.

In 2020, Naito et al. strongly associated α_s1_-casein and β-casein with specific IgE levels, despite their low inherent homology [51]. These authors reported that patients with severe CMA exhibited allergic reactions when products containing casein phosphopeptides (CPP), including chewing gum, topical creams, and toothpaste, were used. CPPS are conserved sequences on α-casein and β-casein, with the “SSSEE” core motif composed of phosphorylated serine residues, and have been reported to have an IgE-binding capacity and basophil activation comparable to that induced by casein in vitro, causing severe allergic symptoms [52]. In Table 1, we summarized the cross-reactive allergens and their epitopes related to cow’s milk and two other livestock products.

In recent years, cow milk–soybean cross-allergy has garnered considerable attention because of their cross-allergic reactions, and both are high-quality, affordable, and readily accessible high-protein foods with high consumption rates in the general population. For example, soybean allergens, including the α-subunit of Gly m 5, the Gly4 subunit of Gly m 6, Gly m Bd 28K, P34, and Gly m Bd 60K, are associated with cross-reactivity between patients allergic to milk and soybean because they recognize CMA-specific IgE antibodies [53]. In 2014, Curciarello et al. [54] identified Gly m5 as cross-reactive with cow milk casein, with later studies revealing cross-reactive epitopes between the soybean protein P34 and cow milk casein [55]. Similarly, in 2016, Candreva et al. [56] identified the soybean cross-reactive allergen protein Gly m Bd 28K using sera from patients with CMA. The protein induced degranulation of human peripheral blood basophils and IL-13 secretion in immunized mice, confirming that soybean-cow milk cross-allergic reactions. In 2017, Candreva et al. [57] investigated peptides from α_s1_-casein and Gly m 5 via enzymatic hydrolysis, reporting shared common sequence regions among four functional epitopes of α_s1_-casein, namely, AA91-100 (YLGYLEQLLR), AA97-110 (QLLRLKKYKVPQLE), AA19-30 (NLLRFFVAPFPE), AA18-1 (ENLVEQPLGQHKIPHKPR), and three functional epitopes of Gly m 5, namely AA125-142 (LRRHKNKNPFLEGSNRFE), AA366-385 (KNPQLRDLDIFLSIVDMNE), and AA526-543 (GNKGRKGPLSSILRAFY). In 2020, another study reported two sets of potential cross-reactive epitopes between cow milk α_s1_-casein and soybean proteins: α_s1_-casein AA49-71 (KEKVNELSKDIGSESTEDQAMED) with Gly m Bd 60k AA319-341 (KEQIRALSKRAKSSSRKTISSED) and α_s1_-casein AA112-130 (QLLRLKKYKVPQLEIVPNS) with Gly m Bd 60k AA164-182 (QLQNLRDYRILEFNSKPNT) [58]. Collectively, these findings confirm the potential cross-reactivity between cow milk and soybean proteins.

**Table 1:** Cross-reactive allergens and their epitope sequences in livestock products. For meat allergies, the broad cross-reactivity of serum albumin has been discussed in [14], including sequence alignments; however, definitive epitope regions have not been identified.

Main Allergen	Allergen	Cross-Linear Epitope	Cross-Allergen	Reference
Egg	Hen’s egg lysozyme	Y_55_DTQAIVQNNDSTEY_69_ and W_79_CKDDQNPHSSNICN_93_	Cow’s milk α-lactalbumin	[46]
	Hen’s egg α-vitellin	C_312_IAEVEKDAIPENLP_326_, F_525_DEKLFTFHADICTLPDTEKQIKKQTALVE_554_, Y520VPKAFDEKLF_530_ and L_555_ LKHKPKATEE_565_	Cow’s milk BSA	
Milk	Cow’s milk β-lactoglobulin	BLG-1 (KPTPEGDLEILLQK), BLG-2 (LQKWENDECAQKKIIAEKTKIPAVFKIDA), BLG-3 (KALKALPMHIRLSFN)	Goat’s milk β-lactoglobulin	[50]
	α-casein	SSSEE	β-casein	[52]
	Cow’s milk α_s1_-casein	AA91-100 (YLGYLEQLLR), AA97-110 (QLLRLKKYKVPQLE), AA19-30 (NLLRFFVAPFPE), AA18-1 (ENLVEQPLGQHKIPHKPR)	Soybean Gly m 5	[57]
	Cow’s milk α_s1_-casein	AA49-71 (KEKVNELSKDIGSESTEDQAMED) and AA112-130 (QLLRLKKYKVPQLEIVPNS)	Soybean Gly m 5	[58]

## 3. Application of Livestock Product Allergens in Diagnosis and Treatment

Recent advances in proteomics, peptide library screening, and bioinformatics have enabled the precise localization and identification of linear epitopes in livestock product allergens [11], deepening our understanding of allergic mechanisms and supporting innovative diagnostic and therapeutic strategies. For example, component-resolved diagnosis using synthesized linear epitope peptides offers significantly improved diagnostic accuracy and specificity, reducing the number of false-negative results associated with the use of natural extracts [12,59].

Linear epitope-based peptide immunotherapy offers enhanced safety by preventing IgE cross-linking and severe allergic reactions, indicating promising desensitization for food allergies [13,14]. The oral immunotherapy approach increases the reaction threshold to a specific food allergen by repeatedly administering food protein to the patient while gradually increasing the dosage over months until a predetermined maintenance dose is reached [60]. With respect to diagnostic and therapeutic methods for soybean-milk cross-reactivity, Candreva et al. [61] synthesized a Gly m 30k-derived peptide with cross-reactive allergen epitopes; its minimal B- and T-cell core sequence was NKIQDKVTIDGY. Furthermore, preventive oral administration of the derived peptide partially alleviated allergic symptoms in milk-allergic Balb/c mice, suggesting that the use of soybean peptides as a cross-reactivity-based strategy for CMA prevention. However, oral immunotherapy using intact allergen proteins poses challenges due to variations in patient-specific immunodominant peptides [62], thereby increasing the cost and limiting its broader applicability.

**Figure 5:**
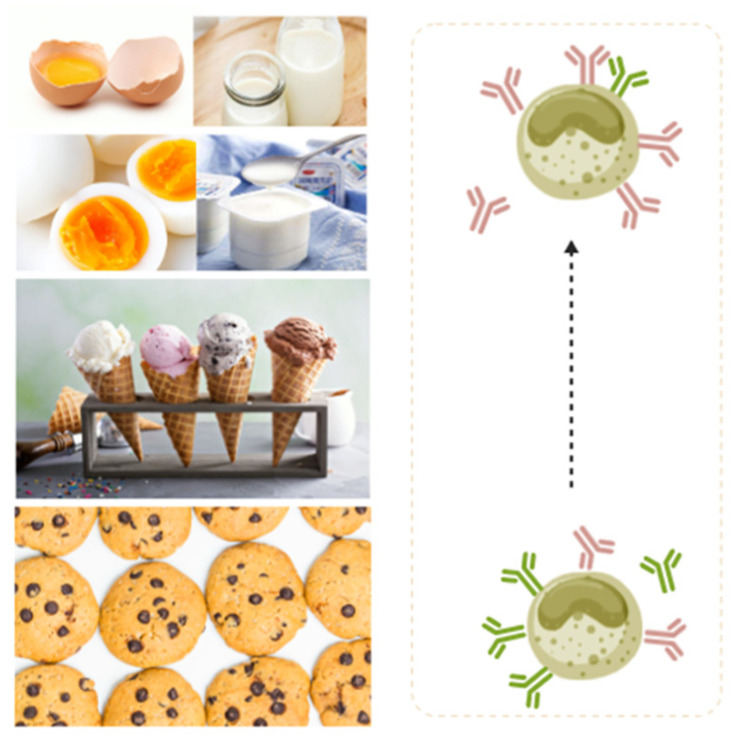
The egg-milk ladder showing changes in the antibody binding patterns with gradual changes. The green antibodies represent specific immunoglobulin E antibodies that may trigger allergic reactions.

Egg–milk ladder desensitization therapy for children allergic to eggs and milk involves gradually introducing allergens into the child’s diet, starting with low-allergenicity forms (e.g., cooked eggs or baked milk goods) and progressing to higher-risk forms (e.g., raw eggs or pasteurized milk) (Figure 5) [63].

Initially, recommended for non-IgE-mediated allergies, the egg–milk ladder method has been applied to IgE-mediated cases [64], with over half of surveyed physicians endorsing its use to treat IgE-mediated allergies [65]. Different countries have developed various egg–milk ladder models [66,67,68]. Notably, its safety in infants and young children with a history of anaphylaxis remains controversial, with some studies reporting successful tolerance in infants with such a history [69]. Nevertheless, researchers still recommend the cautious application of this therapy for this specific patient group, along with the need for parental competence in managing acute allergic reactions. Presently, studies on horizontal comparisons between the egg–milk ladder and other treatment methods, such as oral immunotherapy or allergen immunotherapy, are lacking, warranting further investigation to address this gap. Table 2 summarizes the advantages and disadvantages of these three therapeutic approaches.

**Table 2:** Comparison of the advantages, disadvantages, and application scopes of the food ladder, oral tolerance induction, and allergen immunotherapy.

Method	Advantages	Disadvantages	Application	References
Food ladder	Can be administered at home; Can be discontinued at any time	High demands on parental competency; Difficult to quantify allergen protein doses at each step	Mainly used for infants and children with common food allergies (e.g., egg, milk); particularly suitable for non-IgE-mediated or mild-to-moderate IgE-mediated allergies.	[70]
Oral tolerance	protocol is relatively standardized.	may relapse after discontinuation.	Primarily used for IgE-mediated food allergies (e.g., peanut, milk, egg); applicable to children and some adults.	[60,71]
Allergen immunotherapy	Applicable to a wide range of allergens, effects are well-defined for specific allergens.	Primarily limited to inhalant or insect venom allergens; treatment process can be cumbersome; high cost; Risk of anaphylaxis during treatment	Traditionally used for allergic rhinitis, asthma, and insect sting allergies; for food allergies, still largely investigational (e.g., oral/sublingual immunotherapy).	[72]

## 4. Conclusions and Future Perspectives

Livestock products serve as vital dietary protein sources; however, increasing demand and novel food emergence have increased the associated cross-sensitization risks, increasingly affecting the global population. This review summarizes advances in immunological cross-reactivity research on livestock product allergens, outlining cross-reactivity characteristics and recent linear epitope studies in meat, eggs, and milk. Furthermore, this study highlights the critical role and challenges of linear epitopes in allergy diagnosis and treatment.

Linear epitopes play a central role in cross-allergy research, with many databases providing allergen and epitope sequence queries. Linear epitopes are antigenic determinants composed of continuous amino acid sequences in antigenic proteins, and their immunogenicity does not depend on the three-dimensional folding structure of the protein. Therefore, they retain their activity even after protein hydrolysis and are of great value in the clinical diagnosis of food allergies. The variable region (V region) of IgE antibodies forms high-affinity non-covalent interactions with continuous peptide segments of linear epitopes through their antigen-binding sites (Paratope), involving hydrogen bonding, hydrophobic interactions, and van der Waals forces. Typical linear epitopes usually consist of 4–6 amino acids, such as Ara h 2–15 in the peanut allergen and tropomyosin in tropomyosin [73,74,75]. The primary structure of these sequences directly determines the specificity of IgE recognition. Although their binding affinity is generally lower than that of conformational epitopes, linear epitopes are more easily exposed during digestion and become key targets in persistent food allergies such as peanut and nut allergies. When two or more IgE molecules simultaneously bind to multiple linear epitopes on the same allergen, it triggers the crosslinking of the high-affinity receptor FcεRI on the surface of mast cells or eosinophils, activating Syk tyrosine kinase, which in turn triggers the intracellular signaling cascade of PLCγ, IP3, and Ca^2+^ release, ultimately leading to cell degranulation and the release of mediators such as histamine, trypsin, and leukotrienes, thereby triggering an allergic reaction.

**Figure 6:**
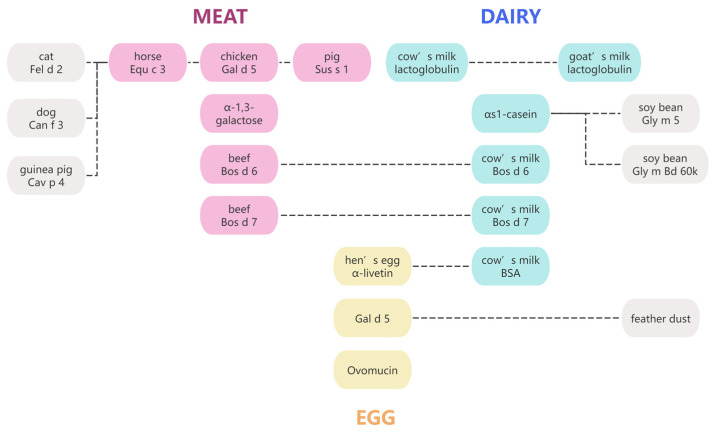
Cross-reactive allergens among livestock products; pink represents meat allergens; blue represents dairy allergens; yellow represents egg allergens; and gray represents allergens from other species. The dashed lines indicate that the connected allergens exhibit cross-reactivity with each other.

However, to the best of our knowledge, no database enables direct searches for grouped cross-reactive allergens and their linear epitopes, which limits cross-allergy research. Notably, frequent inter-species cross-sensitization occurs across livestock products (Figure 6), with meat–dairy cross-allergies being more common than egg allergens, which form a more distinct cluster. Visualizing grouped cross-reactive allergens may enhance the understanding of cross-allergy frequency and patterns across different species. The proposed approach aims to provide a theoretical basis for the establishment and refinement of databases on cross-reactive allergens and epitopes.

Notably, 3D cell culture technology presents a novel perspective regarding cross-reactive allergen research, offering advantages, including more comprehensive in vivo environment simulation and superior specificity compared with those of two-dimensional cultures, thereby reflecting intercellular interactions and cytokine production. These advantages may allow for a more accurate and reliable model for identifying cross-reactive food allergens. These findings may further the understanding of immune response mechanisms, particularly in investigations of the individual binding patterns of IgE and epitopes and subsequent signal transduction. In the future, 3D cell culture technology may facilitate more accurate assessment of immune responses triggered by food allergens, along with distinguishing genuine cross-reactions, identifying potential cross-reactive allergens, and serving as a highly promising tool for allergology research.

Animal studies remain crucial approaches for evaluating the mechanisms of cross-allergic reactions; however, mature animal models for cross-reactive allergens and standardized protocols for assessing associated parameters, such as combined immunization doses and administration methods, are lacking. Different mouse strains have been employed in food allergy research, with Balb/c mice commonly utilized for validating sensitization mechanisms and the occurrence of allergic reactions and C3H/HeJ mice utilized in cross-allergy research involving reaction occurrence and severity. Reportedly, C3H/HeJ mice present changes in IgE levels and exhibit more pronounced clinical allergic symptoms compared with those in Balb/c mice [76]. Similarly, some studies may employ stronger adjuvants or different administration routes, such as intraperitoneal injection or intranasal administration instead of oral intake, to amplify the allergic response in experimental animals. However, the implications of these approaches warrant careful consideration, as stronger clinical signs may help clarify the intensity of cross-reactivity and can affect the perception of cross-allergy severity by failing to mimic the natural human response state.

Substantial progress has been reported in recent years regarding the identification of epitopes in food allergens and the underlying mechanisms of cross-reactivity; nevertheless, many challenges persist. The findings of this study suggest that future research should focus on the following key areas: (1) investigating the differential IgE responses among diverse ethnic and regional populations to elucidate the roles of genetic and environmental factors in allergic reactions; (2) developing precise identification methods for cross-reactive allergens and their linear epitopes; and (3) intensifying research into the mechanisms of immunological cross-reactivity and conducting clinical trials to optimize allergen detection methods and personalized treatment strategies. Although identification techniques such as yeast two-hybrid, co-immunoprecipitation, and fluorescence resonance energy transfer have not been widely applied in allergen detection, they have garnered considerable attention in recent years owing to their significant potential. Furthermore, the development of epitope-specific antibodies and epitope-based vaccines may emerge as a major research focus in the future, offering novel strategies for food allergy management.

## Data Availability

No new data were created or analyzed in this study. Data sharing is not applicable to this article.

## Abbreviations

The following abbreviations are used in this manuscript:

APCs	Antigen-Presenting Cells
Th0	Naive T helper cells
Th2	Type 2 T helper cells.
CMA	Cow-milk allergy
α-Gal	α-1,3-galactose
AGS	α-1,3-galactose (α-Gal) syndrome
OVM	Ovomucin
BLG	β-lactoglobulin
CPP	Casein phosphopeptides
